# The Circadian Clock Coordinates the Tradeoff between Adaptation to Abiotic Stresses and Yield in Crops

**DOI:** 10.3390/biology12111364

**Published:** 2023-10-24

**Authors:** Hang Xu, Yi Zuo, Jian Wei, Lei Wang

**Affiliations:** 1 Key Laboratory of Plant Molecular Physiology, Institute of Botany, Chinese Academy of Sciences, Beijing 100093, China; xuhang18@mails.ucas.ac.cn (H.X.); zuoyi@ibcas.ac.cn (Y.Z.); 2 University of Chinese Academy of Sciences, Beijing 100049, China; 3 Center of Soybean, Jilin Agricultural University, Changchun 130117, China; weijian@jlau.edu.cn; 4 Academician Workstation of Agricultural High-Tech Industrial Area of the Yellow River Delta, National Center of Technology Innovation for Comprehensive Utilization of Saline-Alkali Land, Dongying 257300, China; 5 China National Botanical Garden, Beijing 100093, China

**Keywords:** circadian clock, crop, abiotic stresses, tradeoff, yield

## Abstract

**Simple Summary:**

The circadian clock plays a crucial role in helping plants to effectively acclimate to ever-changing environmental conditions. The proper functioning of the circadian clock is integral to the growth and development of plants. In crops, the circadian clock system exerts a multifaceted influence on yield and the response to abiotic stress. This review provides a comprehensive overview of the mechanisms through which the components of a crop’s circadian clock impact its response to abiotic stress and yield. We propose that the circadian clock may orchestrate the balance between abiotic stresses and yield in crops, which is useful for the future molecular design of crop breeding.

**Abstract:**

Plants have evolved a circadian clock to adapt to ever-changing diel and seasonal environmental conditions. The circadian clock is generally considered an internal system that has evolved to adapt to cyclic environmental cues, especially diel light and temperature changes, which is essential for higher plants as they are sessile organisms. This system receives environmental signals as input pathways which are integrated by circadian core oscillators to synchronize numerous output pathways, such as photosynthesis, the abiotic stress response, metabolism, and development. Extreme temperatures, salinity, and drought stresses cause huge crop losses worldwide, imposing severe pressure on areas of agricultural land. In crop production, the circadian system plays a significant role in determining flowering time and responding to external abiotic stresses. Extensive studies over the last two decades have revealed that the circadian clock can help balance the tradeoff between crop yield-related agronomic traits and adaptation to stress. Herein, we focus on summarizing how the circadian clock coordinates abiotic stress responses and crop yield. We also propose that there might be an urgent need to better utilize circadian biology in the future design of crop breeding to achieve high yields under stress conditions.

## 1. Introduction

From lower organisms to higher living organisms, circadian clocks have evolved continuously to adapt to environmental changes. During its evolution, the circadian clock system has recruited more components to form an intricate, self-sustaining network. For instance, while the circadian clock in green algae has been identified as a simple feedback loop with only two genes, circadian clocks in higher living organisms can be modeled as intricate networks of transcription–translation-based autoregulatory feedback loops [1,2]. The circadian clock system can be reset or entrained by external cues such as light, temperature, and nutrition to synchronize with external environmental factors [3]. Conceptually, a plant’s circadian clock comprises three parts: the input pathway that perceives and receives environmental signals, the core oscillator that is entrained by the input signals and integrates endogenous circadian rhythms, and the output pathways [4]. In the core oscillator network in plants, clock genes/proteins form multiple transcriptional–translational negative feedback loops and maintain an approximately 24 h oscillation [3,4]. The output pathway of the circadian clock covers almost all essential biological processes, including cellular homeostasis, growth, and development, by controlling the transcription of target genes, protein stability, and metabolism [3,4,5]. When faced with abiotic stresses, a disturbance in the circadian clock system causes the rearrangement of partially responsive circadian components, which reorient new pathways to overcome the stresses [5]. As an endogenous and self-sustained time-keeping mechanism, the circadian clock enables plants to predict and anticipate exterior fluctuations using internal physiological and biochemical networks. This greatly improves the fitness of plants [6].

Studies conducted using the model plant Arabidopsis and the circadian systems of crops revealed that they possess similar circadian components [7], with the crops’ circadian components having analogous functions compared to Arabidopsis. This suggests that circadian clocks may be utilized to increase crop yields [8,9]. As a signal integration hub, a plant’s circadian clock ensures its normal growth and reproduction and responds to biotic and abiotic stresses [8]. Consequently, the plant’s circadian system senses and activates downstream stress response signaling pathways when confronted with mild stresses. This causes a redistribution of energy, shortens the growth stage, and accelerates reproduction [10,11].

Notably, recent evidence demonstrates that, in crops, the circadian clock contributes to the coordination of various abiotic stresses and crop yield. The expression of a large proportion of abiotic stress-responsive genes, as well as the downstream signaling pathways of the stress response, are temporally controlled by the circadian clock. Hence, the circadian system plays a role in balancing stress responses and biomass. Here, we mainly focus on how a crop’s circadian system coordinates the tradeoff between responses to abiotic stress and crop yield and how the precise “cost” (the energy required to respond to abiotic stresses) and “return” (crop yield) mechanisms work in crops.

## 2. Circadian Clock Components Regulate Heat and Cold Tolerance in Crops

The security of future crop yields is severely threatened by heat and cold stress. There is a vast amount of evidence that the plant circadian system is linked to environmental temperature stress. Transcriptomic analyses conducted in Arabidopsis revealed that approximately 50% of heat stress-responsive genes and 40% of cold-regulated genes are expressed rhythmically under constant light, indicating that many temperature stress-responsive genes are under the control of the circadian clock [12].

**Figure 1:**
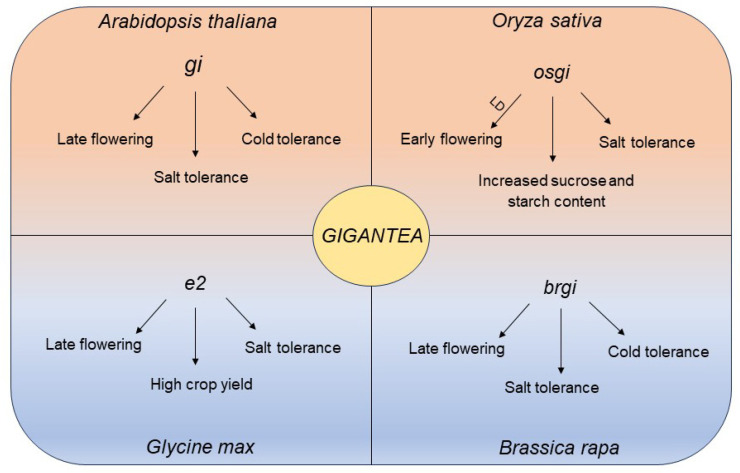
The diverse functions of *GI* in various crops. Specifically, in *Arabidopsis thaliana*, the *gi* mutant exhibited a late-flowering phenotype and demonstrated tolerance to cold and salt stress. In *Oryza sativa*, the *osgi* mutant displayed early flowering under long-day conditions as well as salt tolerance and increased sucrose and starch content. In *Glycine max*, the loss-of-function *GI* homologous gene *E2* was associated with late flowering and high crop yield and exhibited a salt tolerance phenotype. Furthermore, in *Brassica rapa*, the lack of *GI* resulted in similar phenotypes to those observed in the Arabidopsis *gi* mutant, including late flowering and tolerance to cold and salt stress.

In Arabidopsis, the *LUX ARRYHTHMO* (*LUX*) gene encodes a MYB family transcription factor protein, which is a key circadian clock component and has been found to play a positive role in low-temperature stress [13]. It was revealed that CBF1/DREB1b (DRE-binding/C-repeat binding factor) can bind to the *LUX* promoter to regulate the transcription of *LUX* and is, thus, involved in the response to cold stress [14]. In rice, OsLUX was shown to play a positive regulatory role in low-temperature stress. The overexpression of *OsLUX* (*OsLUX*-*OE*) can enhance cold tolerance in the seedling stage, while the knockout of *OsLUX* reduces cold tolerance in seedlings [15]. *OsLUX*-*OE* promoted the expression levels of oxidative stress-related genes, which could subsequently improve the rate of scavenging reactive oxygen species (ROS), thus enhancing tolerance to chilling stress [15]. *GIGANTEA* (*GI*) was first identified via a late-flowering mutant in Arabidopsis, and subsequent studies found that GI acts in response to multiple abiotic stresses, including salt and cold stresses [16,17,18]. Recently, Xie et al. found that a *gi* mutant displays late-flowering and cold-tolerant phenotypes in *Brassica rapa*, which shows great potential for enhancing cold and salt stress tolerance and improving biomass [19] (Figure 1). In addition, the *GI* gene in sweet potato was also found to be involved in multiple stress responses, including cold stress [20].

Emerging transcriptome datasets show that the transcriptional level in response to heat stress is quite dynamic [21,22]. In Arabidopsis, the transcript abundance of core clock genes is altered in response to heat stress during the day [21]. In the cereal crop barley (*Hordeum vulgare*), the transcript levels of circadian clock core genes, including *CIRCADIAN CLOCK ASSOCIATED 1* (*CCA1*), *GI*, *PSEUDO RESPONSE REGULATOR 59* (*PRR59*), *PSEUDO RESPONSE REGULATOR 73* (*PRR73*), *PSEUDO RESPONSE REGULATOR 95* (*PRR95*), and *LUX,* are increased at higher temperatures. In addition, the response of *GI* and *PRR* genes to temperature stress is lost in the *elf3* mutant, indicating that their response to temperature cues may be dependent on ELF3 [23]. In soybean, heat stress causes a milder perturbation in the global circadian rhythm [24]. In rice, among the 25 known heat shock transcription factors (HSFs), 17 show rhythmic expression under diel conditions, while 11 of those 17 are induced by heat stress [25,26]. These datasets indicate a close relationship between the circadian clock and heat responses, although the direct mechanisms of crop circadian clock component responses to heat stress are not yet fully understood.

**Figure 2:**
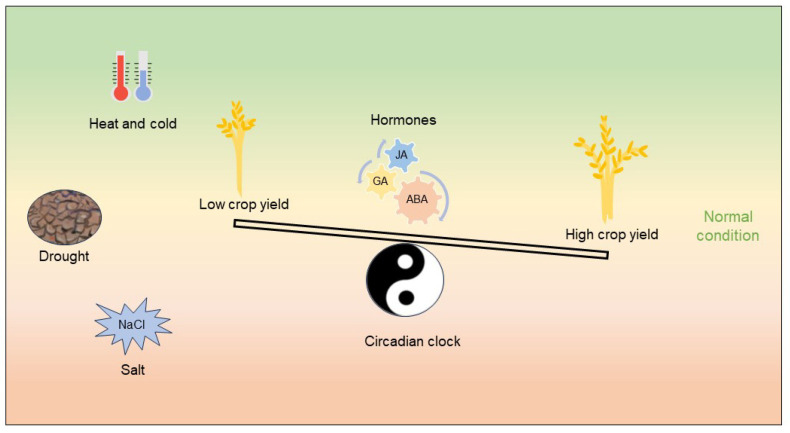
The circadian clock of crops plays a crucial role in managing the balance between adapting to abiotic stress and achieving optimal yield. This process is facilitated by the feedback regulatory effect of the circadian system and hormones, which work in tandem to maintain homeostasis in crops. The self-sustaining and stable nature of the circadian system further contributes to this process.

High- and low-temperature stress bring about a decreased crop yield; here, we enumerate the evidence of how circadian clock components respond to high and low temperatures. During the process of circadian system resistance to temperature stresses, normal growth stages are frequently disturbed. The circadian clock maintains relative homeostasis in rearranging limited energy to coordinate the tradeoff between stress responses and crop yield to a certain extent (Figure 2).

## 3. Crop Circadian Clock Coordinately Regulates Salt Stress and Flowering Time

The increase in saline–alkali land areas due to global warming has intensified the shortage of agricultural land and, therefore, developing crops with better adaptability to high-salt conditions has become an urgent priority. Circadian clock components have been shown to function as important integrators at multiple levels in response to salt stress. In Arabidopsis, the triple mutant *d975* (lack of core clock genes *PRR5*, *PRR7*, and *PRR9*) is more tolerant to high-salinity, cold, and drought stresses than the wild type [27]. Further study revealed that *d975* initiates a stress response by mediating cyclic expression of stress response genes, including *DREB1/CBF* [27]. However, the detailed underlying mechanism of PRR5/7/9 in salt stress tolerance remains to be fully explored.

**Figure 3:**
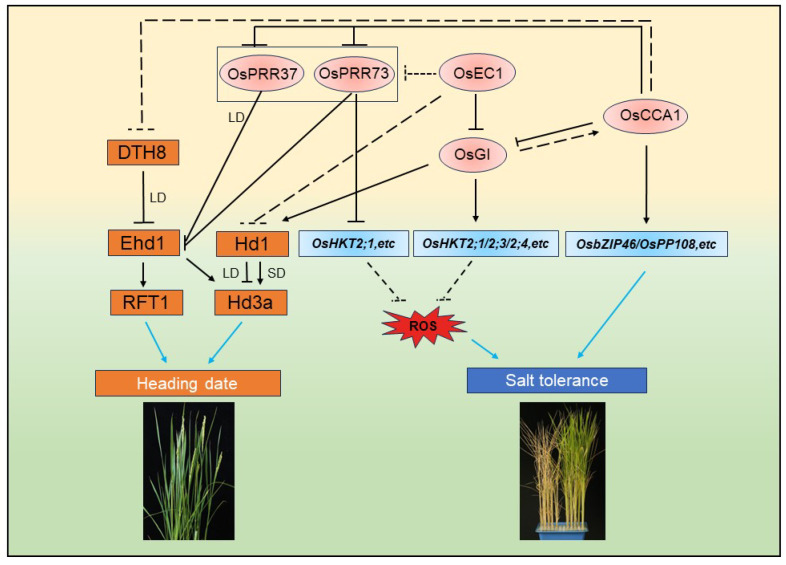
A proposed model for the role of rice circadian clock in regulating salt tolerance and heading date. OsCCA1 acts as a flowering activator by repressing the expression of *DTH8* and *OsPRR37*, finally leading to the upregulation of florigens (*Hd3a* and *RFT1*). OsPRR73 and OsGI both function in regulating salt tolerance by modulating downstream *OsHKTs*. OsEC1 also plays a vital role in both the salt stress response by the OsGI–OsHKTs module and flowering time regulation through the Hd1–Hd3a/RFT1 pathway. In addition, there is a transcription–translation feedback loop in circadian clock components. Arrows and bars indicate upregulation and downregulation. Solid and dashed lines represent direct and indirect regulations. Blue lines show the effect on the final result in rice.

Nevertheless, in rice, OsPRR73 was demonstrated to function as a positive regulatory factor in salt stress. OsPRR73 confers salt tolerance by interacting with histone deacetylase 10 (HDAC10), thus forming a transcription-repressive complex to inhibit the transcription of *high-affinity K^+^ transporters 2;1* (*HKT2;1*) and maintain cellular Na^+^ homeostasis [28]. Meanwhile, it was found that OsPRR73 acted as a rice floral inhibitor, since overexpression of *OsPRR73* resulted in a late-flowering-time phenotype under both long-day (LD) and short-day (SD) conditions [29]. Mechanistically, OsPRR73 binds to the *early heading date 1* (*Ehd1*) promoter and represses its expression [29]. Thus, it is conceivable that OsPRR73 plays critical roles in coordinating rice heading date control and salt stress tolerance (Figure 3).

Another core circadian clock component, OsCCA1, was also identified as a key integrator in the salt stress response by affecting the abscisic acid (ABA) signaling pathway and rice heading date control. Specifically, OsCCA1 binds to the promoters of *OsPP2C* (*protein phosphatase 2C*) members and *OsbZIP46* (*basic region and leucine zipper 46*) to activate their transcription, thus affecting the salt response [30]. Moreover, OsCCA1 was identified as a floral inducer. In the japonica cultivar “Dongjin”, the T-DNA insert mutant *oscca1* caused late flowering under both long-day (LD) and short-day (SD) conditions [31]. Importantly, three nonsynonymous SNPs in *OsCCA1* were identified. Rice varieties harboring japonica-type *OsCCA1* alleles had an earlier flowering time than those harboring indica-type *OsCCA1* alleles [31]. Therefore, OsCCA1 is a critical component in balancing abiotic stress tolerance and heading date, which jointly determines rice yield (Figure 3).

Recently, rice circadian clock complex Evening Complex 1 (OsEC1), composed of OsELF4a, OsELF3-1, and OsLUX, was shown to be needed for salt tolerance and flowering time regulation, thus deeply affecting crop yield. To withstand salt stress, OsEC1 directly binds to the promoter of *OsGI* to repress its expression and transmits signals via downstream Na^+^- and K^+^-related transporters OsHKT2;1/2;3/2;4 [32]. In addition to the salt-sensitive phenotype, null mutants of *OsEC1* also exhibit a late flowering time compared with the wild type. For the regulation of flowering time, OsEC1 mainly functions through the classical OsGI–Hd1–Hd3a pathway under both SD and LD conditions. In addition, OsEC1 might also negatively regulate flowering time independent of the OsGI pathway by repressing the expression of *OsPRR73* and *Heading date 1* (*Hd1*) [32]. Hence, OsEC1 could coordinately work in the regulation of salt response and heading date. The multiple functions of OsEC1 suggest that circadian clock components could maintain the balance between stress tolerance and crop yield on a large scale.

Interestingly, as a dual target in the control of both the heading date and salt stress response of OsEC1, OsGI also has dual identity in coordinating this tradeoff. Unlike the *gi* mutant, which caused an extremely late-flowering phenotype in Arabidopsis, the null mutants of *OsGI*, the rice homolog of *GI*, displayed an early-flowering-time phenotype in both LD and nLD (natural LD) conditions [32,33]. In addition, the *osgi-101* mutant displayed a salt tolerance phenotype compared to the wild type [32] (Figure 1), indicating that OsGI plays an important role in coordinating salt stress tolerance and heading date.

The salt overly sensitive (SOS) pathway is essential for maintaining Na^+^ homeostasis in plant cells [34,35]. Recently, it was shown that the SOS pathway has close reciprocal regulation with the clock component GI. On the one hand, SOS1, the plasma membrane Na^+^/H^+^ antiporter in Arabidopsis, could function as a salt-dependent circadian clock regulator via the clock component GI. SOS1 could directly interact with the GI protein in a salt-dependent manner to stabilize it, thus maintaining a proper circadian period under salt stress conditions [36]. On the other hand, GI conveys salt tolerance through SOS2, which is an SNF1-related protein kinase. GI could physically interact with SOS2. In the absence of salt stress conditions, the GI-SOS2 complex blocks SOS2 to activate SOS1. Under saline conditions, GI is degraded, thus subsequently releasing SOS2. Furthermore, the released SOS2 interacts with SOS3 to form a salt-responsive complex and then activates SOS1, thus positively regulating the salt stress response [37]. Similarly, the homolog members of SOS pathways in rice are likely also influenced by the circadian clock. It was shown that the transcript levels of *OsSOS3* and *OsSOS2* exhibited rhythmic and diel expression patterns, while *OsSOS1* did not [38]. To date, there is little evidence linking the rice circadian clock with SOS pathway members, but existing clues have pointed to possible close ties. Notably, GI is a unique protein in both Arabidopsis and rice and plays multiple functions in both abiotic stress tolerance and flowering time control. Therefore, it would be of great interest to investigate whether the reciprocal interaction between the SOS pathway and GI protein occurred in rice and whether the underlying mechanisms are conserved. Moreover, transcriptomic profiles show that the circadian clock regulates nearly 80% of all genes in rice [39]. Many studies have shown that numerous salt-responsive genes are regulated by the circadian clock. For example, the receptor for activated C kinase 1 (RACK1), a WD40-type protein, is regulated by the rice circadian clock at both the transcriptional and translation levels and plays an important role in the salt stress response [40].

In soybean, *Juvenile* (*J*) is known to be an ortholog of the Arabidopsis EC component *ELF3*, and loss-of-function *J*-alleles lengthen soybean maturity and enhance grain yield [41]. *J* is a direct transcriptional repressor of the legume-specific flowering repressor *E1*, and the J protein physically associates with the *E1* promoter to reduce its transcription, relieving the repression of two important *FLOWERING LOCUS T* (*FT*) genes, *FT2a* and *FT5a,* thus promoting flowering under SD conditions [41]. Apart from the modulation in flowering time, Cheng et al. found that the expression of *J* was induced by NaCl treatment. Under salt stress, J could positively regulate the expression of downstream stress-responsive genes and convey salt tolerance [42], suggesting that the *J* gene has a dual role in flowering time control and salt stress tolerance. Soybean *E2*, an ortholog of Arabidopsis *GI*, has a negative effect on both crop yield and salt stress. The *e2* single mutants showed earlier flowering times and high grain yields in high-latitude regions [43]. After salt stress, the *e2* mutant displayed a salt tolerance phenotype by releasing peroxidase and scavenging ROS [44] (Figure 1). Therefore, the soybean clock components J and E2 may serve as molecular hubs to coordinate salt tolerance and flowering time. The different genetic combinations of J and E2 may be used for breeding elite cultivars for regional adaptation.

Recently, a high-temporal-resolution transcriptomic analysis in foxtail millet demonstrated that more than 40% of drought responsive genes (DRGs) were affected at specific time points, either ZT0 or ZT16, suggesting that crosstalk between drought stress and the circadian clock also occurs in foxtail millet [45]. Hence, it might be a rapid and convenient way to edit the clock components in major crops to generate salt-tolerant alleles with proper flowering time to guarantee crop yield under saline conditions.

## 4. Crop Circadian Clock Components Regulate Drought Stress

In recent decades, drought stress has become a severe abiotic stress for crop production. To resist water deficit, plants experience various molecular and physiological changes induced by drought stress. Emerging evidence shows that the circadian clock plays a crucial role in the drought-responsive process. Specifically, it mediates the transmission of drought stress signals, particularly via the ABA pathway [46,47].

**Figure 4:**
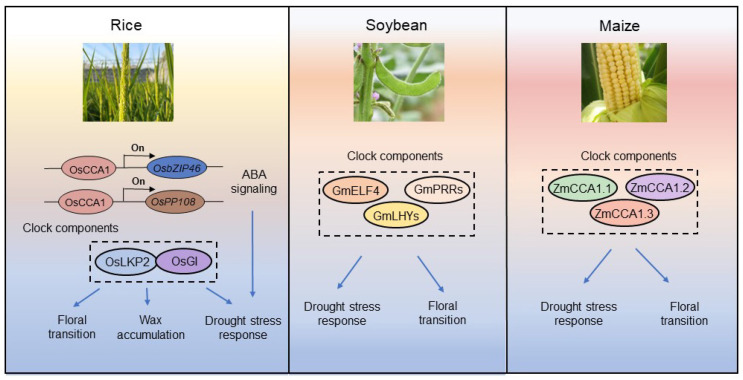
A proposed model for the role of circadian clock components in regulating drought stress response in soybean, rice, and maize. Drought stress repressed the expression of the soybean clock genes *GmELF4*, *GmPRRs*, and *GmLHYs.* In addition, these components also affect floral transition. The rice circadian component OsCCA1 responds to multiple stress responses by involving the ABA signaling pathway. The interaction of the clock components OsLKP2 and OsGI affects downstream processes, including wax accumulation and drought stress responses. In maize, the ZmCCA1 splice variants ZmCCA1.1, ZmCCA1.2, and ZmCCA1.3 have functions in regulating drought tolerance. Arrows and bars indicate upregulation and downregulation. Blue lines show the effect on the final result in rice.

In rice, the clock component OsCCA1 confers drought tolerance via the ABA signaling pathway [30]. Low drought treatment at the early stage in rice can induce early flowering and reduce tiller numbers due to the accumulation of abscisic acid (ABA). In turn, accumulated ABA has feedback effects on the circadian clock and circadian clock-related flowering determination, involving changes in related genes, including *TIMING OF CAB EXPRESSION1* (*OsTOC1*), *grain number*, *plant height*, *heading date 7* (*Ghd7*), and *phytochrome B* (*PhyB*) [48]. Apart from these genes, other flowering-related genes, such as *OsGI*, *OsELF3*, *OsPRR37*, and *OsMADS50*, also regulate the flowering process through an ABA-independent pathway [48]. In addition, among the homologs of the Arabidopsis ZEITLUPE family, LOV KELCH REPEAT PROTEIN 2 (OsLKP2) specifically responds to drought stress at the transcript level. The null mutations of OsLKP2 displayed enhanced drought tolerance by increasing cuticular wax biosynthesis, which subsequently inhibited nonstomatal water loss. Furthermore, it was found that OsLKP2 could physically interact with another clock component, OsGI, in the nucleus, and the null mutation of OsGI also showed enhanced drought tolerance with a high density of wax crystals, suggesting that the interaction of OsGI and OsLKP2 negatively regulates wax accumulation to decrease rice resilience to drought stress [49] (Figure 4). This evidence jointly suggests that rice clock components are important for regulating drought tolerance, either in ABA-dependent or ABA-independent pathways.

In soybean, drought stress reduces the expression of circadian clock genes, including *LCL1-*, *GmELF4-*, and *PRR-*like genes [24,50] (Figure 4). Soybean clock components and circadian clock-regulated genes together function to resist drought stress. Soybean drought-responsive genes, including *DREB-*, *bZIP-*, *GOLS-*, *RAB18-*, and *Remorin-*like, change their diel rhythms significantly after exposure to drought conditions [50]. Remarkably, soybean circadian LHY-CCA1-LIKE orthologs, *GmLCLa1*, *GmLCLa2*, *GmLCLb1*, and *GmLCLb2*, have important roles in the drought stress response. Under drought stress, the *GmLCL* quadruple mutant displayed reduced leaf water loss compared with the wild type. Interestingly, the peak expression of *GmLCL* genes was also delayed by drought stress, indicating that there is complex feedback between GmLCLs and drought stress [51]. Upon drought stress, the *GmLCL* quadruple mutant increased the expression of ABA signaling-related genes, such as *PYRABACTIN RESISTANCE-LIKE 17* (*PYL17*), *cytochrome P450 707A* (*CYP707A*), *ABA INSENSITIVE 2* (*ABI2*), and *SNF1-related protein kinase2s* (*SnRK2s*), to address drought tolerance [51]. In addition, amplitude and phase shifts were discovered in *GmPRR7* and *GmTOC1* genes after drought stress [52]. Significant splicing in the *GmPRR3* gene was found under drought conditions. *GmPRR3* may work together with *GmPRR7* and *GmTOC1* to achieve drought responses [52]. Similarly, it was found that two GmLHYs, the orthologs of CCA1/LHY in Arabidopsis, negatively regulate drought stress in soybean through the ABA signaling pathway [53]. A later study found that loss-of-function of *PRR* homologs *GmPRR3b* delayed growth and floral transition [54], which indicates that clock components may coordinately regulate flowering time and drought stress in soybean.

Collectively, circadian clock components may be conserved in coordinating salt tolerance and flowering time control in crops, including but not limited to rice and soybean. In maize, three *ZmCCA1* splice variants were identified in the tropical line CML288, which are predicted to encode three different protein isoforms, namely ZmCCA1.1, ZmCCA1.2, and ZmCCA1.3 [55]. Drought stress induced the expression of *ZmCCA1* to increase tolerance to drought stress. Three types of ZmCCA1 splice variants, ZmCCA1.1, ZmCCA1.2, and ZmCCA1.3, may function differently in response to drought conditions (Figure 4). *ZmCCA1.1* may be a dominant gene in resisting drought tolerance, whereas *ZmCCA1.2* and *ZmCCA1.3* have a relatively minor effect [55]. It may be of great interest to systematically generate maize clock mutants by genome editing to further extensively investigate the role of maize clock components in the regulation of drought stress tolerance in the future.

Many studies have shown that the circadian clock could regulate drought stress via ABA signaling pathways. It is well known that ABA biosynthesis is tightly controlled by the circadian clock in Arabidopsis and that the ABA content in leaves follows a diel oscillation, rising during the day and declining at night [56,57,58]. The ABA content plays a crucial role in determining the final crop yield under drought conditions. Additionally, it was revealed that the ABA content is involved in determining floral transition [59]. Therefore, it is conceivable that the circadian clock plays a critical role in controlling ABA biosynthesis and signaling pathways. Together, the role of the circadian clock in drought stress tolerance, in an ABA-independent way, collectively balances plant development and drought stress tolerance, which ultimately determines crop yield.

## 5. Conclusions and Perspective

The circadian clock of crops performs numerous crucial functions that aid in adapting to environmental stresses and determining yields. Evidence suggests that there is a close relationship between the circadian system and responses to abiotic stresses, such as temperature stress, salt stress, and drought stress. The circadian system functions in both global rhythms and partial components to coordinate stress responses and determine crop yield. Specific regulatory mechanisms involve close collaboration between transcriptional and posttranscriptional modification factors and other modification factors. The circadian clock plays the role of a “clever trader” in precisely allocating finite resources and harmonizing the entire plant to ensure complete reproduction.

However, many details regarding the interaction between circadian clock signals and stress responses remain to be solved. First, plant tissues have their own relatively independent circadian systems [60], and stress responses involving the circadian clock have tissue-specific characteristics. For instance, osmotic stress in barley roots alters the expression of circadian clock genes in shoots [61]. ABA in the phloem and SAM have different roles: drought stress-induced ABA accumulation increases the expression of *GI* and other florigen genes, while ABA at the shoot apex represses flowering [62]. The existence of two seemingly contradictory regulatory modules proves the tradeoff functions of the circadian system. However, more details about how crops respond to multiple abiotic stresses are absent, especially considering the different growing locations of different grains. Second, the interactions between different signaling factors depend on functional diversity in both the plant circadian clock and stress responses. For instance, the circadian clock involving ABA signaling in drought response also includes the functional role of light integration [63]. Moreover, heat stress often accompanies drought stress, which challenges circadian system work efficiency and increases the complexity of regulatory networks. Metabolic feedback and retrograde signaling also showed a high degree of participation in both circadian clock modulation and abiotic stress-responsive processes, while details and applications in crops are lacking. Third, cross-talk between various hormones and the circadian system in simultaneously resisting abiotic stresses needs to be further explored. When salt stress appears, the contents of ABA and gibberellin in plants fluctuate to relieve pressures [64]. Combined with former tissue-specific hormone reactions, the regulatory networks of various combinations of hormones in different tissues are waiting to be replenished. The dynamics of the circadian clock system, hormone content, and abiotic stress responses all have pairwise feedback effects. The tradeoff between the circadian system and stress tolerance is accomplished via hormone signaling to a large extent. Fourth, whether the natural section of the crop circadian clock locus also plays a vital role in coordinating crop yield determination and abiotic stress responses remains unknown. Natural alleles in circadian genes such as *OsCCA1* affect the rice heading date [31], while the differences in their functions in stress tolerance are worth researching. Currently known natural SNPs tend to be linked with photoperiod sensitivity and temperature variation. In the future, more valuable alleles of circadian clock genes should be identified, especially in harmonizing both crop yield and stress tolerance.

Creating crop varieties that combine the virtues of both high yield and certain stress resistance is the aim of plant breeders. In the crop circadian clock system, many genes have the potential to realize this target, such as the *GI* gene. In soybean, loss-of-function of *GI* orthologs *E2* results in earlier flowering, high grain yield, and salt tolerance [43,44]. *GI* provides an ideal target and excellent allelic combinations for the molecular breeding of high-yield and salt-tolerant cultivars in soybean. In this review, we only summarize the circadian rhythm involving abiotic stresses, including heat stress, cold stress, salt stress, and drought stress, to find the balanced relationship between stress responses and crop yield. However, we still missed many other stress types, such as UV-B light stress, mineral stress, and flooding stress, because of the few studies on crop circadian clock systems involving stress responses. In summary, our review offers a new perspective on crop circadian systems and their coordination of the tradeoff between stress adaptation and yield. We hope our understanding of the crop circadian system will aid in future molecular design breeding efforts.

## Data Availability

Data are contained in the article.
